# Preparation, Antidermatophyte Activity, and Mechanism of Methylphloroglucinol Derivatives

**DOI:** 10.3389/fmicb.2018.02262

**Published:** 2018-11-02

**Authors:** Lianbao Ye, Pengfei Lin, Wenjun Du, Yuanyuan Wang, Chunping Tang, Zhibin Shen

**Affiliations:** ^1^School of Pharmacy, Key Laboratory of New Drug Discovery and Evaluation of Ordinary Universities of Guangdong Province, Guangdong Pharmaceutical University, Guangzhou, China; ^2^School of Traditional Chinese Medicine, Guangzhou Key Laboratory of Construction and Application of New Drug Screening Model Systems, Guangdong Pharmaceutical University, Guangzhou, China

**Keywords:** methylphloroglucinol derivatives, preparation, antidermatophyte activity, molecular simulation, antidermatophyte mechanism

## Abstract

In this study a variety of phloroglucinols were isolated from the plant, and the activity experiment showed that the phloroglucinols had strong antifungal activity, especially methylphloroglucinol derivatives such as aspidin PB, dryofragin, aspidinol, aspidin BB, aspidin AB, and albicanol, in which the hydroxyl group of methylphloroglucinol is the active group of compounds, and C-2 or C-6 is the active site. The introduction of different groups in this position could change the properties and bioactivity of the compounds. In this study, different functional groups were introduced to the structure of methylphloroglucinol to obtain methylphloroglucinol derivatives that were synthesized, and antidermatophyte activities on *Trichophyton rubrum, Trichophyton mentagrophytes, Microsporum canis*, and *Gypsum microspore bacteria* were evaluated. Molecular docking verified its ability to combine the protein binding site. The antidermatophyte mechanism of compounds on cytochrome P450 sterol 14a-demethylase, squalene epoxidase, and β-1,3-glucan synthase was investigated by the enzyme-linked immunosorbent assay. The results showed that compounds had an inhibitory effect on four kinds of common dermatophytes in varying degrees, in which compound **g** had the strongest activities, the binding mode of methylphloroglucinol and its derivatives were similar to those of three enzymes, and compounds **e** and **g** had significant effects on the activity of the three enzymes, and compound **g** had a slightly stronger effect than the blank group. Compounds **e** and **g** also had a significant effect on the ergosterol synthesis of *M. canis*. This study could supply some antidermatophyte leading structure and possible mechanism for studying and developing new antifungal agents.

## Introduction

Fungi on skin are widely distributed in nature and frequently present as pathogens in the animal and plant kingdoms (Ruhnke et al., 2011). In recent decades, despite progress in antidermatophyte therapy, fungal infections on skin have remained a major global health concern, due to the development of antifungal drug resistance (Tsui et al., 2016; Shrestha et al., 2017). However, the emergence of resistance to antifungal drugs by diverse pathogenic fungal strains has resulted in an increase in demand for new antifungal agents (Araj et al., 2015). The widely used clinical antifungals are azoles, polyenes, allylamines, and echinocandins. These antifungal scaffolds are known to function by different mechanisms (Kathiravan et al., 2012; Xie et al., 2015). Currently, main targets of the antifungal agents are cytochrome P450 sterol 14a-demethylase (CYP51), squalene epoxidase (SE), and β-1,3-glucan synthase, in which azole and triazole drugs are CYP51 inhibitors widely used as antifungal, antibiotics, and antimycobacterial drugs, spinosins are β-1,3-glucan synthase inhibitors, and allylamine agents act on SE (Huang et al., 2009; Gil-Alonso et al., 2016; Mazu et al., 2016; Yang and Xu, 2016).

*Dryopteris fragrans* was mainly used as a folk medicine at present. It has been found that the main active compounds of *D. fragrans* have a significant effect on a variety of skin diseases caused by fungi (Wan et al., 2014; Zhao et al., 2014; Wang et al., 2015; Du et al., 2016; Huang et al., 2016). Our group isolated a variety of phloroglucinols from the plant, and the activity experiment showed that the phloroglucinols had strong antifungal activity, especially methylphloroglucinol derivatives such as aspidin PB, dryofragin, aspidinol, aspidin BB, aspidin AB, and albicanol, in which the hydroxyl group of methylphloroglucinol is the active group of compounds, and C-2 or C-6 is the active site (Sun et al., 2013; Li et al., 2014; Gao et al., 2016). The introduction of different groups in this position may change the properties and bioactivity of the compounds (Yang, 2017; Lin et al., 2018). In this study, different functional groups were introduced to the structure of methylphloroglucinol to obtain new methyl phloroglucinol derivatives that were synthesized, and antidermatophyte activities on *Trichophyton (T.) rubrum, Trichophyton (T.) mentagrophytes, Microsporum (M.) canis*, and *Gypsum (G.) microspore (M.) bacteria* were evaluated. Molecular docking verified its ability to combine the protein binding site. The antidermatophyte mechanism of compounds on CYP51, SE, and β-1,3-glucan synthase was investigated by the enzyme-linked immunosorbent assay (ELISA), and the effect of compounds **e** and **g** on the ergosterol synthesis of *M. canis* was investigated by ultra performance liquid chromatography (UPLC). This study can supply some antidermatophyte leading structure and possible mechanism for studying and developing new antifungal agents, and it can give foundation of the development of new antifungal drugs of independent intellectual property rights.

## Results and Discussion

### Chemistry

**FIGURE 1:**
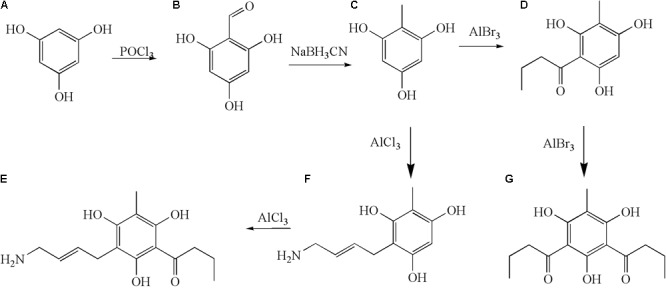
Synthetic route of target compounds.

In order to study the antidermatophyte activities of methylphloroglucinol derivatives, we synthesized **c** analogs to explore the correlation of antidermatophyte activity. We used a divergent strategy to introduce structurally different linkers on C-2 or C-6 of the methyl phloroglucinol moieties. Our group isolated a variety of phloroglucinols from the plant, and the activity experiment showed that the phloroglucinols had strong antifungal activity, such as aspidin PB, dryofragin, aspidinol, aspidin BB, aspidin AB, and albicanol, and the C-6 position of these compounds has the C_1_–C_4_ acyl group. In this study, we introduced butyryl and antifungal pharmacophore allylamine into the methylphloroglucinol and/or kept the C_1_–C_4_ acyl group in the C-6 position using pseudoaspidinol as a lead compound to obtain novel phloroglucinol derivatives **d–g.** The synthetic route of the compounds is shown in Figure 1. The methylphloroglucinol was successfully synthesized using 1,3,5-hydroxybenzene (**a**) as raw material by the Vilsmeier–Haack reaction and reduction reaction. As a result, four derivatives, 2-butyryl-4-methylbenzene-1,3,5-triol (**d**), 2,6-dibutyryl-4-methylbenzene-1,3,5-triol (**e**), (*E*)-2-(4-aminobut-2-enyl)-4-methylbenzene-1,3,5-triol (**f**), and (*E*)-6-(4-aminobut-2-enyl)-2-butyryl-4-methylbenzene-1,3,5-triol (**g**) were obtained with the yield of 28.6, 57.2, 35.7, and 81.2%, respectively.

#### 2-Methylbenzene-1,3,5-triol (**c**)

Yield: 78%, white solid, Mp: 231–234°C, ^1^H nuclear magnetic resonance (NMR) (500 MHz, CDCl_3_): δ (ppm) 10.29 (s, 1H, 4-OH), 9.68 (s, 2H, 2-OH, 6-OH), 5.76 (s, 2H, H-3, 5), 1.80 (s, 3H, H-7). ^13^C NMR (125 MHz, CDCl_3_): δ (ppm) 160.0, 160.0, 157.9, 102.9, 96.4, 96.4, 27.1. EI-MS: 141.14 [M+H]^+^. Anal. calcd for C_7_H_8_O_3_ (140.05): C, 59.99; H, 5.75; O, 34.25; Found: C, 59.94; H, 5.72; O, 34.34.

#### 2-Butyryl-4-methylbenzene-1,3,5-triol (**d**)

Yield: 28.6%, white solid, Mp: 161–164°C, ^1^H NMR (500 MHz, CDCl_3_): δ (ppm) 10.18 (s, 1H, H-2OH), 9.89 (s, 1H, H-4OH), 9.21 (s, 1H, H-6OH), 5.63 (s, 1H, H-5), 2.97 (s, 2H, *J* = 5.5, H-9), 2.03–2.09 (m, 2H, H-10), 1.86 (s, 3H, H-7), 1.17–1.20 (m, 3H, H-11).^13^C NMR (125 MHz, CDCl_3_): δ (ppm) 187.6, 161.0, 161.7, 156.9, 105.1, 103.7, 90.6, 46.0, 27.3, 18.2, 13.7. EI-MS: 209.71 [M+H]^+^. Anal. calcd for C_11_H_14_O_4_ (210.09): C, 62.85; H, 6.71; O, 30.44; Found: C, 62.89; H, 6.70; O, 30.41.

#### 2,6-Dibutyryl-4-methylbenzene-1,3,5-triol (**e**)

Yield: 47.2%, yellow solid, Mp: 260–263°C, ^1^H NMR (500 MHz, CDCl_3_): δ (ppm) 10.18 (s, 1H, 2-OH), 9.89 (s, 1H, 4-OH), 9.21 (s, 1H, 6-OH), 2.97 (s, 4H, *J* = 5.5, H-9, H-13), 2.03–2.09 (m, 4H, H-10, H-14), 1.86 (s, 3H, H-7), 1.17–1.20 (m, 6H, H-11, H-15).^13^C NMR (125 MHz, CDCl_3_): δ (ppm) 187.6, 187.6, 161.0, 161.7, 156.9, 105.1, 103.7, 90.6, 46.0, 46.0, 27.3, 18.2, 18.2, 13.7, 13.7. EI-MS: 279.88 [M+H]^+^. Anal. calcd for C_15_H_20_O_5_ (280.13): C, 64.27; H, 7.19; O, 28.54; Found: C, 64.30; H, 7.13; O, 28.57.

#### (E)-2-(4-Aminobut-2-enyl)-4-methylbenzene-1,3,5-triol (**f**)

Yield: 81.2%, brown solid, Mp: 282–285°C, ^1^H NMR (500 MHz, CDCl_3_): δ (ppm) 10.08 (s, 1H, 2-OH), 9.81 (s, 1H, 4-OH), 9.11(s, 1H, 6-OH), 5.92–5.98 (m, 2H, H-9, H-10), 5.53 (s, 1H, H-5), 1.80 (s, 3H, H-7), 1.69–1.79 (m, 4H, H-8, H-11). ^13^C NMR (125 MHz, CDCl_3_): δ (ppm) 160.1, 159.7, 156.1, 128.6, 114.5, 104.9, 104.1, 90.5, 43.0, 26.5, 19.2. EI-MS: 210.32 [M+H]^+^. Anal. calcd for C_11_H_15_NO_3_ (209.11): C, 63.14; H, 7.23; N, 6.69; O, 22.94; Found: C, 63.10; H, 7.24; N, 6.75; O, 22.91.

#### (*E*)-6-(4-Aminobut-2-enyl)-2-butyryl-4-methylbenzene-1,3,5-triol (**g**)

Yield: 48.7 %, brown solid, Mp: 385–388°C, ^1^H NMR (500 MHz, CDCl_3_): δ (ppm) 9.98 (s, 1H, 2-OH), 9.71 (s, 1H, 4-OH), 9.17(s, 1H, 6-OH), 5.81–5.91 (m, 2H, H-9, H-10), 2.91 (s, 2H, *J* = 5.5, H-13), 2.13–2.19 (m, 2H, H-14), 1.80 (s, 3H, H-7), 1.69–1.79 (m, 4H, H-8, H-11), 1.07–1.19 (m, 3H, H-15). ^13^C NMR (125 MHz, CDCl_3_): δ (ppm) 187.6, 160.1, 159.7, 156.1, 128.6, 114.5, 105.1, 103.7, 90.6, 46.0, 43.0, 27.3, 26.5, 18.2, 13.7. EI-MS: 280.89 [M+H]^+^. Anal. calcd for C_15_H_21_NO_4_ (279.15): C, 64.50; H, 7.58; N, 5.01; O, 22.91; Found: C, 64.53; H, 7.56; N, 4.98; O,22.93.

### *In vitro* Antidermatophyte Assay

In order to investigate their potential as antidermatophyte agents, according to the CLSI M38-A2 Reference Method for Antifungal Susceptibility Testing, the microdilution method was used to investigate the antidermatophyte activities of methylphloroglucinol derivatives **b**–**g** along with those for two known antifungal drugs [terbinafine hydrochloride (TBF), miconazole nitrate (MCZ)] on four dermatophytes (*T. rubrum, T. mentagrophytes, M. canis, G. M. bacteria*). The minimal inhibitory concentrations (MICs) and minimal fungicidal concentrations (MFCs) of compounds against the four dermatophytes are shown in Table 1. The MIC was defined as the lowest concentration that completely inhibited visible fungal growth in the wells after 7 days of incubation, and the MFC was indicated by the well that showed no growth after culturing on the Sabouraud dextrose agar medium. Since triazole drug MCZ and allylamine agent TBF are widely used as antifungal, respectively, act on CYP51 inhibitors and SE, we chose MCZ and TBF as positive control. Cell suspensions (200 μL) containing 0.156–160 μg/μL of compounds were added to the wells of a 96-well microtiter plate and incubated for 48 h at 35°C. The six compounds showed an inhibitory effect on the four dermatophytes in varying degrees. Because compounds had lower antidermatophyte activities than the positive control group, antidermatophyte activities were comparative between compound groups. The strong and weak order of antidermatophyte activities were as follows: **g-e-b-d-c-f**, in which compound **g** has the strongest antidermatophyte activities, especially on *M. canis* with the MIC value of 10 μg/mL; the inhibitory effect of compounds **c** and **f** is not obvious. It was worth noting that these compounds had strong inhibition on *T. mentagrophytes* and *M. canis*. The MIC values of **e** and **g** on the four dermatophytes were very close, especially on *T. mentagrophytes* and *M. canis*. Compounds **e** and **g** had stronger inhibition on *M. canis.* So we chose *M. canis* to perform time-kill curve assay research.

**Table 1:** Minimal inhibitory concentrations (MICs) and MFCs of compounds against four dermatophytes.

	*Trichophyton rubrum* (μg/mL)		*Trichophyton mentagrophytes* (μg/mL)		*Gypsum microspore bacteria* (μg/mL)		*Microsporum canis* (μg/mL)	
	MIC	MFC	MIC	MFC	MIC	MFC	MIC	MFC
**b**	125	250	>160	>160	62.5	125	30	30
**c**	>160	>160	>160	>160	>160	>160	>160	>160
**d**	125	250	62.5	62.5	250	250	125	125
**e**	160	160	20	20	20	20	20	20
**f**	>320	>320	>320	>320	>320	>320	>320	>320
**g**	60	80	20	20	20	20	10	10
TBF	0.16	0.32	0.05	0.25	0.25	0.5	0.25	0.5
MCZ	0.25	0.25	0.25	0.5	0.5	1	0.5	1

*MCZ, miconazole nitrate; TBF, terbinafine hydrochloride.*

### Time-Kill Curve Assays

**FIGURE 2:**
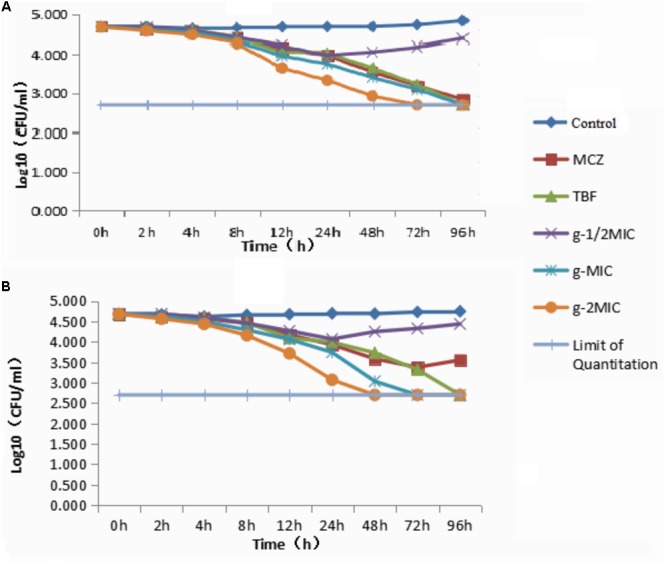
Time-kill curve: **(A)** Time-kill curve of compound **e** against *Microsporum canis*; **(B)** time-kill curve of compound **g** against *M. canis.* (The limit of quantitation indicated that there was no colony growth on plate medium.)

In order to understand the rate of the antidermatophyte activity, compounds **e** and **g** were selected for time-kill assays against the *M. canis owing to* stronger inhibition of compounds **e** and **g** on *M. canis.* The time-kill curve of compounds **e** and **g** was as shown in Figure 2. The colonies of different time points in Time-Kill Curve were seen in Supplementary Material. Compounds **e** and **g** against *M. canis* were investigated by the colony counting method. The limit of quantitation indicated that there was no colony growth on plate medium. The results showed that the two derivatives could inhibit the growth of *M. canis* for a certain period of time, and when the drug concentration was less than MIC, the two derivatives could inhibit the growth of *M. canis.* The difference was that when the drug concentration was between MIC and 2MIC, compound **g** was stronger than compound **e** to kill *M. canis*.

### Effect of Compounds **e** and **g** on Ergosterol Synthesis of *M. canis*

**FIGURE 3:**
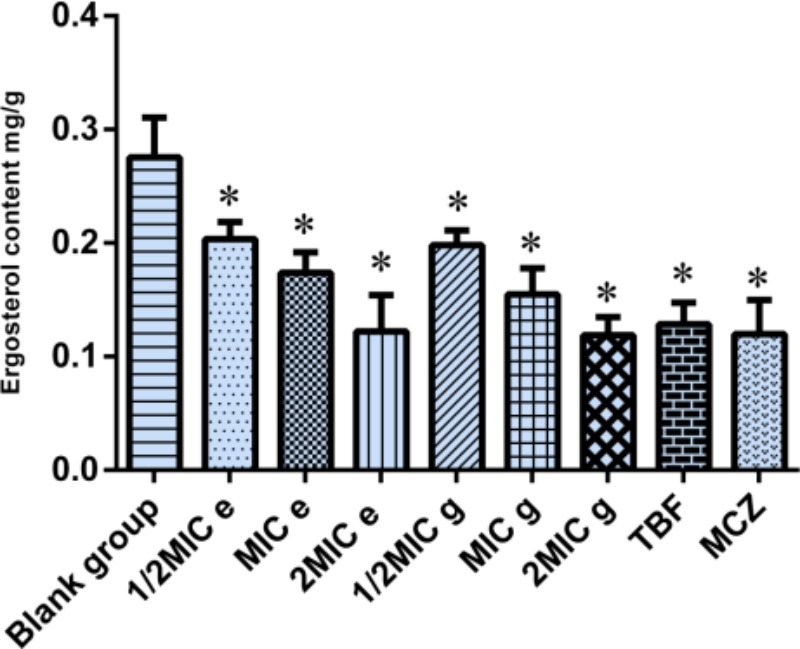
Effect of compounds **e** and **g** on the ergosterol synthesis of *Microsporum canis* (blank group, ergosterol; MIC ***e***-value, 20 μg/mL; MIC ***g***-value, 10 μg/mL; MCZ, miconazole nitrate; TBF, terbinafine hydrochloride; ^∗^comparison with blank group, *P* < 0.05).

In a previous study, we found that active composition of *D. fragrans (L.)* Schott influenced the cell wall to inhibited growth of *T. rubrum* and *T. mentagrophytes* by inhibiting β-1,3-glucan synthase, which is the key kinase of ergosterol synthesis. So we determined ergosterol content of *M. canis* by UPLC to investigate the effect of compounds **e** and **g** on the ergosterol synthesis of *M. canis* before and after administration. The results were as shown in Figure 3. Compared with the blank group, the ergosterol content in *M. canis* decreased significantly after administration. When the concentration of compound **g** was 1/2MIC, MIC, and 2MIC, the decreased rate of ergosterol content was 15.76, 34.28, and 56.83%, respectively, which was higher than those of compound **e** with the decreased rate of 13.55, 26.17, and 55.59%. The decreased rate of ergosterol content in MIC groups of TBF and MCZ was 45.37 and 49.11%, respectively, as seen in Table 2.

**Table 2:** Effects of different concentrations of drugs on ergosterol in *Microsporum canis.*

Group		Ergosterol content (mg/g)	Decrease rate of ergosterol content (%)
Blank group		0.2754 ± 0.085	–
Compound **e**	1/2MIC	0.2035 ± 0.015^∗^	13.55
	MIC	0.1738 ± 0.018^∗^	26.17
	2MIC	0.1223 ± 0.032^∗^	55.59
Compound **g**	1/2MIC	0.1983 ± 0.013^∗^	15.76
	MIC	0.1547 ± 0.023^∗^	34.28
	2MIC	0.1189 ± 0.016^∗^	56.83
	TBF	0.1286 ± 0.019^∗^	45.37
	MCZ	0.1198 ± 0.030^∗^	49.11

*MIC ***e****-value, 20 μg/mL; MIC****g***-value, 10 μg/mL; MCZ, miconazole nitrate; TBF, terbinafine hydrochloride. ^∗^Comparison with blank group, P < 0.05.*

### Effect of Compounds **e** and **g** on CYP51, SE, and β-1,3-Glucan Synthase

**FIGURE 4:**
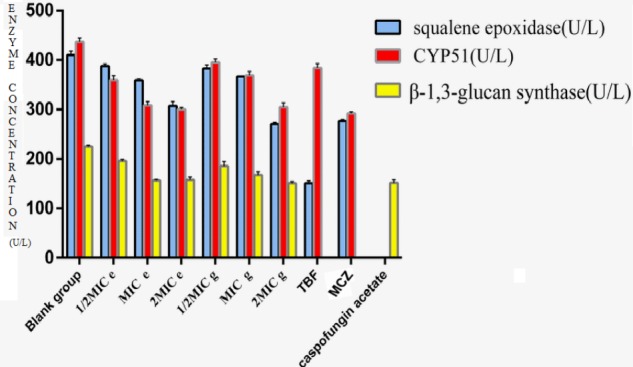
Effect of compounds **e** and **g** on CYP51, squalene epoxidase, and β-1,3-glucan synthase. (MIC ***e***-value, 20 μg/mL; MIC ***g***-value, 10 μg/mL; MCZ, miconazole nitrate; TBF, terbinafine hydrochloride.)

The effect of compounds **e** and **g** on the ergosterol synthesis of *M. canis* showed that compounds **e** and **g** could inhibit the synthesis of ergosterol (Table 3). CYP51 and SE are key enzymes of ergosterol synthesis. So we deduced that methylphloroglucinol derivatives should inhibit ergosterol synthesis by inhibiting CYP51 and SE to affect the formation of fungal cell membrane. Currently, the main targets of the antifungal agents are CYP51, SE, and β-1,3-glucan synthase. In the present study, the effects of compounds **e** and **g** on CYP51, SE, and β-1,3-glucan synthase were investigated by ELISA using TBF, MCZ, and caspofungin acetate as positive control. The results are shown in Figure 4. The results showed that compounds **e** and **g** had significant inhibitions (*P* < 0.01) on three enzymes when the concentration of the compounds was 2MIC, and compound **g** had a slightly stronger effect than the blank group and stronger inhibition than compound **e**. With the increase in the concentration of the compounds, their activities against the three target enzymes decreased in a dose-dependent manner. According to the results of compounds **e** and **g** on the ergosterol synthesis of *M. canis*, we could deduce through the antifungal mechanism that compounds **e** and **g** made the ergosterol content in *M. canis* decrease by inhibiting the activities of CYP51 and SE, which resulted in synthesis failure of the cell film, blocking the synthesis of the fungal cell wall by inhibiting β-1,3-glucan synthase.

**Table 3:** Effect of compounds **e** and **g** on squalene epoxidase, CYP51, and β-1,3-glucan synthase.

Group		Drug concentration (μg/mL)	Squalene epoxidase (U/L) (X ± SD)	CYP51(U/L) (X ± SD)	β-1,3-Glucan synthase (U/L) (X ± SD)
Blank group		–	410.36 ± 7.7	437.39 ± 6.58	224.82 ± 2.82
Compound e	1/2MIC	10	387.65 ± 4.68^∗^	359.97 ± 8.16^∗^	196.10 ± 3.28^∗∗^
	MIC	20	359.37 ± 2.01^∗^	308.46 ± 7.75^∗^	156.62 ± 1.77^∗∗^
	2MIC	40	307.25 ± 8.75^∗∗^	300.25 ± 3.87^∗∗^	157.87 ± 5.64^∗∗^
Compound g	1/2MIC	5	382.77 ± 7.37^∗^	395.04 ± 7.39^∗^	185.50 ± 9.09^∗∗^
	MIC	10	366.62 ± 0.53^∗^	368.81 ± 7.94^∗^	167.86 ± 5.78^∗∗^
	2MIC	20	270.1 ± 3.89^∗∗^	304.99 ± 8.51^∗∗^	150.85 ± 3.64^∗∗^
TBF		0.25	151.06 ± 4.74^∗∗^	383.98 ± 8.9^∗^	∖
MCZ		0.5	276.89 ± 7.47^∗∗^	292.34 ± 3.31^∗∗^	∖
Caspofungin acetate		0.25	∖	∖	151.95 ± 5.72^∗∗^

*MIC ***e****-value, 20 μg/mL; MIC****g***-value, 10 μg/mL; MCZ, miconazole nitrate; TBF, terbinafine hydrochloride; ^∗^comparison with blank group, P < 0.05; ^∗∗^comparison with a blank group, P < 0.01.*

### Docking Studies

**FIGURE 5:**
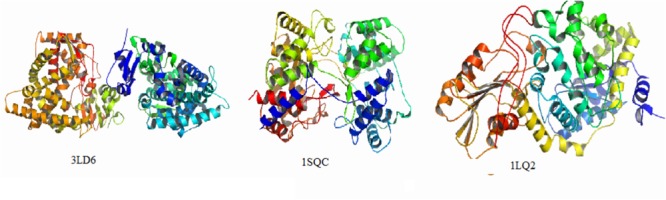
Enzyme crystal complexes of 3LD6, 1SQC, 1LQ2.

Currently, the main targets of the antifungal agents are CYP51, SE, and β-1,3-glucan synthase, in which azole and triazole drugs are CYP51 inhibitors widely used as antifungal and antimycobacterial activity, spinosins are β-1,3-glucan synthase inhibitors, and allylamine agents act on SE (Huang et al., 2009; Gil-Alonso et al., 2016; Mazu et al., 2016; Yang and Xu, 2016). In this study, we investigated the interaction of compounds with protein sites of kinases. The X-ray crystallographic structures of CYP51 (PDB ID: 3LD6), SE (PDB ID:1SQC), and β-1,3-glucan synthase (PDB ID: 1LQ2) were taken from PDB^1^. Enzyme crystal complexes are shown in Figure 5.

**FIGURE 6:**
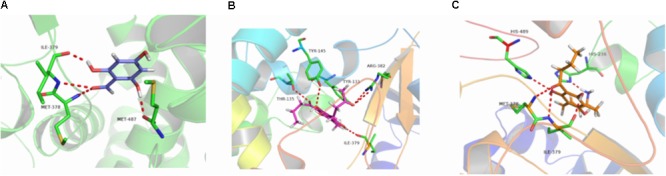
3D docking conformation of compounds with 3LD6: **(A)** 3D docking conformation of compound **b** with 3LD6; **(B)** 3D docking conformation of compound **e** with 3LD6; **(C)** 3D docking conformation of compound **g** with 3LD6.

**FIGURE 7:**
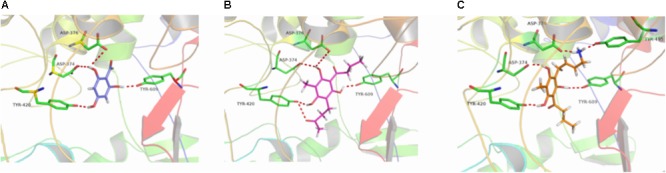
3D docking conformation of compounds with 1SQC: **(A)** 3D docking conformation of compound **b** with 1SQC; **(B)** 3D docking conformation of compound **e** with 1SQC; **(C)** 3D docking conformation of compound **g** with 1SQC.

**FIGURE 8:**
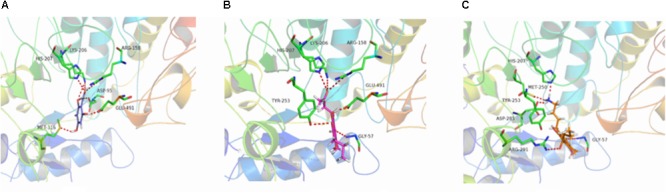
3D docking conformation of compounds with 1LQ2: **(A)** 3D docking conformation of compound **b** with 1LQ2; **(B)** 3D docking conformation of compound **e** with 1LQ2; **(C)** 3D docking conformation of compound **g** with 1LQ2.

The binding energies of complexes between each compound and the active sites of the receptor are shown in Table 4. Compounds **e** and **g** had higher binding energies in accordance with the results of antidermatophyte activities, which further confirmed inhibition of compounds **e** and **g** on CYP51, SE, and β-1,3-glucan synthase. It is noteworthy that compound **b** had stronger inhibition on *M. canis* but lower binding energies. Docking conformations of compounds **b, e**, and **g** with the active sites of protein 3LD6, 1SQC, and 1LQ2 are shown in Figures 6–8, respectively. Results of docking showed that the binding modes of compounds to protein sites of CYP51, SE, and β-1,3-glucan enzyme were similar, which interacted through hydrogen bonding formed by H or O in compound molecule and O or H in amino acid, hydrophobic interaction and Pi–Pi interaction between the benzene ring and the TRP amino acid group. All compounds could combine with TRP34, MET316, GLU491, MET250, LYS206, TYR253, TRP286, GLY57, and PHE144. The differences were that compound **b** could combine with SER55, LEU54, GLY56, and MET250, compound **e** could combine with TRP266, HIS207, HOH848, and HOH869, and compounds **g** could combine with SER59, ASP285, MET316, LEU38, and GLU36, which could cause different activities of compounds. The binding effect of compounds **e** and **g** was stronger than that of compound **b**, which could be related to the butyryl and allylamino groups.

**Table 4:** -CDOCKER energy of compounds to three enzymes.

	–CDOCKER energy		
Receptor		
Compounds	3LD6	1SQC	1LQ2
Compound **b**	17.57	23.66	31.57
Compound **c**	17.69	26.2	34.45
Compound **d**	24.01	32.57	39.27
Compound **e**	30.59	40.18	43.65
Compound **f**	22.43	32.65	34.81
Compound **g**	31.09	41.92	42.95
TBF	17.93	40.68	35.67
MCZ	22.02	33.79	35.66

*MCZ, miconazole nitrate; TBF, terbinafine hydrochloride.*

**FIGURE 9:**
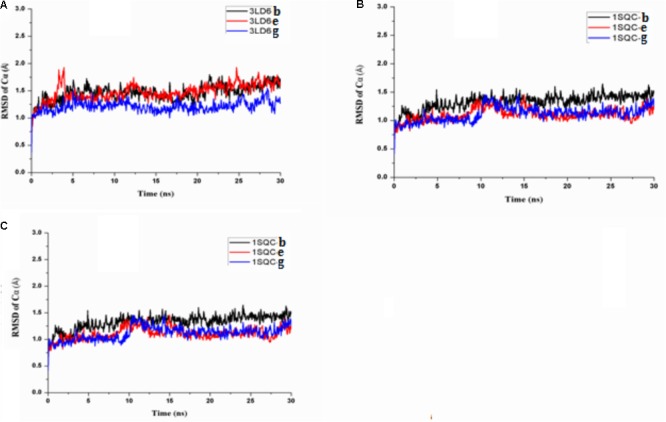
Molecular dynamics simulation of compounds with 3LD6, 1SQC, and 1LQ2: **(A)** molecular dynamics simulation of compounds with 3LD6; **(B)** molecular dynamics simulation of compounds with 1SQC; **(C)** molecular dynamics simulation of compounds with 1LQ2.

In order to explore the interaction of compounds with proteins, we carried out molecular dynamics, and the results are shown in Figure 9. The results of molecular dynamics showed that the root-mean-square deviation (RMSD) values of the three compounds remained 1.0–1.5 for the three enzyme kinetic simulations within 30 ns, which indicated the rationality and stability of the molecular docking between the three compounds and 3LD6, 1SQC, and 1LQ2. The bind energies of compounds to the three enzymes are shown in Table 5. The binding energies of compounds **e** and **g** were higher than that of compound **b**, which were consistent with the results of antidermatophyte activities.

**Table 5:** The binding energy of compounds to three enzymes.

	Binding energy		
	Compound e	Compound g	Compound b
1LQ2	–6.67	–7.04	–5.95
1SQC	–4.74	–5.16	–4.50
3LD6	–3.65	–4.68	–3.42

## Experimental Details

### General Methods of Synthesis

The solvents and reagents were purchased from commercial vendors and were dried and purified by conventional methods before use. NMR spectra were recorded on a Bruker AC-300P spectrometer with tetramethylsilane (TMS) as the internal standard and CDCl_3_ as solvent. ESI mass spectra were performed on an API-3000 LC-MS spectrometer. TLC analysis was carried out on silica gel 60 F_254_ silica plates (Merck, KGaA, Germany).

#### Synthesis of b

Phosphorus oxychloride (875 μL) was added to a solution of 1,3,5-hydroxybenzene (a) (500 mg, 3.97 mmol) and dimethylformamide (725 μL) in ethyl acetate (15 mL), stirred for 2 h at room temperature, and filtered by vacuum. Solvent was evaporated to obtain the crude product, which was used for the next step.

#### Synthesis of c

To the solution of 1-formyl-2,4,6-trihydroxy benzene (b) (1.54 g, 10 mmol) in tetrahydrofuran (20 mL), we added methyl orange (1–2 drops) followed by the addition of sodium cyanoborohydride (1.25 g, 20 mmol). The pH of the reaction mixture was maintained at 4.0. The reaction mixture was stirred for 12 h at room temperature and then extracted with ethyl acetate. The organic layer was washed with brine solution and finally dried over Na_2_SO_4_. Solvent was evaporated to obtain the crude product, which was purified by column chromatography with hexane/EtOAc.

#### Synthesis of d and e

The mixture of c (640 mg, 4.6 mmol), CS_2_ (10 mL), AlCl_3_ (3.1 g, 13.8 mmol), and nitrobenzene (8 mL) was refluxed for 1 h, and then n-butyryl chloride (10 mmol) was added. The mixture was continuously stirred for 3 h. The reaction was detected by TLC (EtOAc/petroleum = 1/1). The reaction solution was cooled to room temperature and was adjusted to pH 7 with HCl and extracted with EtOAc. Solvent was evaporated to obtain the crude product, which was purified by column chromatography with EtOAc/petroleum (1/3) to obtain d and e.

#### Synthesis of f

The mixture of c (100 mg, 9.2 mmol), CH_2_Cl_2_ (30 mL), and AlCl_3_ (4.6 g, 18.4 mmol) was refluxed for 1 h and was added to a solution of 4-chloro-2-buten amine (975 mg, 9.2 mmol) in CH_2_Cl_2_ and stirred for 12 h at room temperature and then treated with 10% hydrochloric acid. The organic layer was isolated and distilled under reduced pressure to remove CH_2_Cl_2_, and then was extracted with acetone. Solvent was evaporated to obtain the crude product, which was purified by column chromatography with EtOAc/petroleum (1/1) to obtain f.

#### Synthesis of g

Compound g was prepared in analogy to compound d.

### *In vitro* Antifungal Assay

#### Antifungal Agents

A 16 mg/mL stock solution of compounds was prepared in dimethyl sulfoxide (DMSO) and stored at -20°C in the dark (foiled wrapped). The antifungal agents fluconazole (FLC, A434403, 99%) and TBF (M0T26A, >98%) were obtained from Shouguang Fukang Pharmaceutical Inc. and Melone Pharmaceutical Inc. FLC and TBF were dissolved in normal saline at a final concentration of 2 mg/mL and were stored at -20°C. Compounds were mixed with potato dextrose agar (PDA).

#### Organisms and Culture Conditions

*Trichophyton rubrum* CMCC(F)T1a, *T. mentagrophytes* CMCC(F)T5a, *G. M. bacteria* MCC(F)M2C, and *M. canis* CMCC(F)Td were obtained from Institute of Dermatology, Chinese Academy of Medical Sciences (Nanjing). These dermatophytes were *Candida parapsilosis*. Filamentous fungi and dermatophytes were cultivated at 35°C in Roswell Park Memorial Institute (RPMI) 1640 medium (with L-glutamine, without sodium bicarbonate; Sigma-Aldrich, St. Louis, MO, United States) buffered to a pH of 7.0 with 0.165 M morpholinepropanesulfonic acid buffer (Sigma-Aldrich).

#### MIC Value Determination by the *in vitro* Antifungal Assay

Disks of dermatophytes were obtained from cultures and were aseptically inoculated onto the center of each Petri plate with extract and control sets. The Petri plates were incubated at 35°C for 7 days. Fungal conidia were prepared by inoculating the fungal culture in normal saline (0.01% Tween-80) and were adjusted to 1 × 10^4^–3 × 10^4^ CFU/mL. Then, they were distributed uniformly on PDA medium by sterilizing cotton swab. Sterile paper disks (6.8 mm) were impregnated with compounds and placed on the culture plates. The MICs and MFCs were determined by the broth micro dilution assay. MCZ and TBF as the positive control of this experiment were used after shaking; 100 mL of the solution was added to the wells of 96-well plates. Cell suspensions (200 μL) containing 0.156–160 μg/μL of compounds were added to the wells of a 96-well microtiter plate and incubated for 48 h at 35°C. The MIC was defined as the lowest concentration that completely inhibited visible fungal growth in the wells after 7 days of incubation. MFC was indicated by the well that showed no growth after culturing on PDA medium (CLSI, 2008).

### Time-Kill Curve Assays

In order to understand the rate of the antidermatophyte activity, compounds **e** and **g** were selected for time-kill assays against *M. canis* owing to stronger inhibition of compounds **e** and **g** on *M. canis.* The efficacy of the compounds to eliminate *M. canis* was evaluated using a published protocol (Nishad et al., 2016). The time-kill curve took shape according to the plate colony counting method. Dermatophyte cells were incubated in PDA medium overnight; one part was taken out to achieve an OD_600_ of 0.20, and the other part (100 μL) was added to RPMI 1640 medium in sterile culture tubes. MCZ, TBF, and compounds **e** and **g** were then added to the solution to achieve drug concentrations at 1/2MIC, MIC, and 2MIC and total volumes in each culture tube to 1.8 mL. The tubes were then incubated at 35°C with continuous agitation (200 rpm) for 0, 2, 4, 8, 12, 24, 48, 72, and 96 h. The second part was removed from each solution and serially diluted in sterile ddH_2_O. Each dilution was then spread onto PDA plates and incubated at 35°C. Colonies were then enumerated after 24–48 h of incubation. The experiments were performed in triple.

### Effect of Compounds **e** and **g** on the Ergosterol Synthesis of *M. canis*

*Microsporum canis* were cultured on PDA medium for a week at 32°C and 90% relative humidity and prepared to a concentration of 1.5 × 10^8^ CFU/mL with sterile saline. To a 250 mL sterile flask, the 100 mL of RPMI 1640 medium was added to reach a concentration of 1 × 10^4^ CFU/mL and cultured at 30°C for 3 days. The 5 mL fungal suspensions were added to a sterile centrifuge tube, followed by the addition of the drug as follows: control group, 1/2MIC compound **e**, MIC compound **e**, 2MIC compound **e**, 1/2MIC compound **g**, MIC compound **g**, 2MIC compound **g**, 2MIC terbinafine, 2MIC miconazole, 2MIC caspofungin, cultured at 30°C for 3 days. Each was performed for three times. The supernatant was removed by centrifugation (3000 rpm, 3 min); 10 mL of sterile distilled water was added, vortexed for 2 min, and repeated three times. The 0.3 g of hyphae was added to 2 mL of the prepared 25% KOH solution and heated for 1 h at 85°C, cooled to room temperature, and extracted with petroleum ether (10 mL × 3). The organic layer was evaporated to dryness. Moderate MeOH was added to the residue and sonicated for 10 min and was set to 5 mL. The ergosterol content in *M. canis* was investigated by UPLC (He et al., 2014).

### Effect of Compounds **e** and **g** on CYP51, SE, and β-1,3-Glucan Synthase

The effects of compounds **e** and **g** on CYP51, SE, and β-1,3-glucan synthase were investigated by ELISA using TBF, MCZ, and caspofungin acetate as positive control. The experiment was carried out according to a published protocol (Brian et al., 2012). Activities of compounds were measured in a polymerization assay in 96-well format. The experiment was divided into three groups: control group (pure enzyme), positive control group (pure enzyme plus positive drug), compound **e** groups (pure enzyme plus compound **e**) (1/2MIC, MIC, and 2MIC), and compound **g** groups (pure enzyme plus compound **g**) (1/2MIC, MIC, and 2MIC). There were three parallel wells in the blank control group and sample group and 2 parallel wells in the standard control group. The 50 μL of distilled water was added to each well and incubated for 30 min at 37°C. The reaction was stopped by the addition of 100 μL of 20% trichloroacetic acid. Plates were chilled for a minimum of 10 min, and washed with five cycles of water (about 1 mL/well each cycle) using a Packard Filtermate Harvester. Scintillation fluid (40 μL/well) (Packard ULTIMA GOLD TM-XR) was added and the sealed plates were counted in a WALLAC BETA counter in top-counting mode at an efficiency of approximately 40%.

### Docking Studies

#### Molecular Docking

Molecular docking was performed with the CDOCKER program that was interfaced with Discovery Studio 2.5.5. The X-ray crystallographic structures of CYP51 (PDB ID: 3LD6), SE (PDB ID: 1SQC), and β-1,3-glucan synthase (PDB ID: 1LQ2) were taken from PDB (see text footnote 1) and were used as protein structures. Briefly, the ligands were docked into the corresponding protein’s binding site complied with the protocol, which was generated by ligands from the crystal structures of 3LD6, 1SQC, and 1LQ2 with random hydrogen atoms and GasteigereHückel charges but not water, and other parameters were default values except that the threshold was 1. The structures of receptors were minimized to 10,000 cycles using the Powell method in DS 2.5.5. The geometries of all compounds were optimized by the conjugate gradient method of TRIPOS. The convergence criterion was identified as 0.001 kcal/mol.

#### MD Simulations

The molecular dynamics simulations were performed on the basis of molecular docking using AMBER 9.0 for ligands and AMBER ff03 for protein, using Gaussian 09 soft to calculate electrostatic potential, and using Antechamber/RESP/ESP to obtain atomic charge. The program selected TIP3P water model and added Na^+^ to maintain electrical neutrality. The heating operation was carried out from 0 to 300 K in 100 ps using Langevin dynamics at a constant volume, which included 2500 cycles of steepest descent minimization, followed by 2500 cycles of conjugated gradient minimization. Finally, periodic boundary conditions of 30 ns were performed for the whole system with a normal pressure of 1 atm and a normal temperature of 300 K in the production step.

## Conclusion

Six methylphloroglucinol derivatives were successfully synthesized. The phenolic hydroxyl group of the phloroglucinols was the active group of compounds. 2,6-Dibutyryl-4-methylbenzene-1,3,5-triol (**e**) and (*E*)-6-(4-aminobut-2-enyl)-2-butyryl-4-methylbenzene-1,3,5-triol (**g**) showed stronger antidermatophyte activities than other compounds. The antidermatophyte mechanism was that compounds made the ergosterol content in *M. canis* decrease by inhibiting the activities of CYP51 and SE, which resulted in synthesis failure of the cell film, blocking the synthesis of the fungal cell wall by inhibiting β-1,3-glucan synthase. The docking experiment showed that compounds **e** and **g** had higher binding energies in accordance with the results of antidermatophyte activities, which further confirmed inhibition of compounds **e** and **g** on CYP51, SE, and β-1,3-glucan synthase. This study can supply some antidermatophyte leading structure and possible mechanism for studying and developing new antifungal agents, and it can give foundation of the development of new antifungal drugs of independent intellectual property rights.

## Author Contributions

ZS and LY contributed to the conception and design of the experiments, analysis of the data, and revision of the paper. PL selected and analyzed the data. YW and CT performed the biological studies. WD performed the docking studies.

## Conflict of Interest Statement

The authors declare that the research was conducted in the absence of any commercial or financial relationships that could be construed as a potential conflict of interest.

## Footnotes

1 www.rcsb.org/pdb
