# Supplementary material for: Preparation, Antidermatophyte Activity, and Mechanism of Methylphloroglucinol Derivatives
Source: Front Microbiol. 2018 Nov 2;9:2262. doi: 10.3389/fmicb.2018.02262 (PMC6224349; doi:10.3389/fmicb.2018.02262)
Supplement: Supplementary file 1 [file Image_1.pdf]

Title: Preparation, Anti-dermatophyte Activity and Mechanism of Methylphloroglucinol Derivatives

Authors: Lianbao Ye, Wenjun Du, Pengfei Lin, Yuanyuan Wang, Chunping Tang, Zhibin Shen

Supplementary information

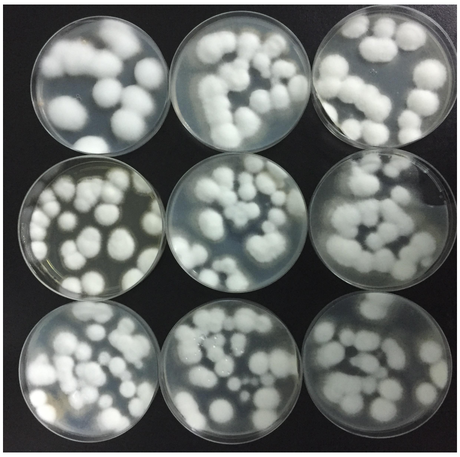

0 h after administration

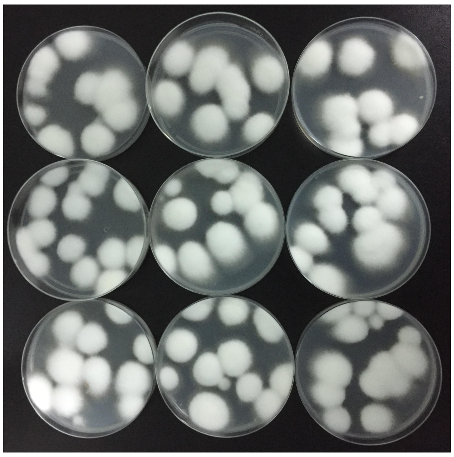

1 h after administration

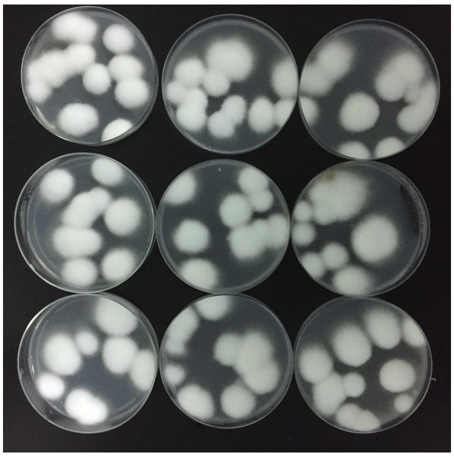

2 h after administration

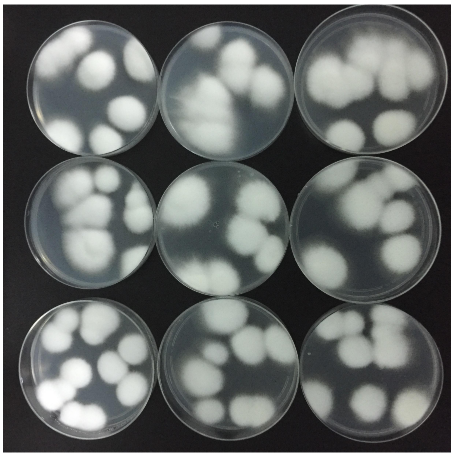

4 h after administration

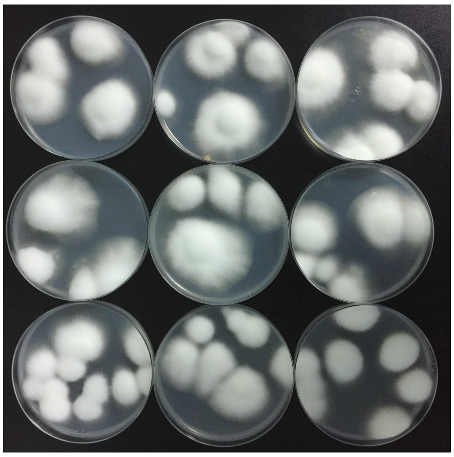

8 h after administration

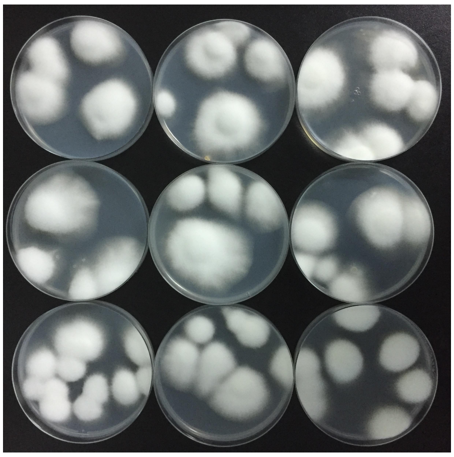

12 h after administration

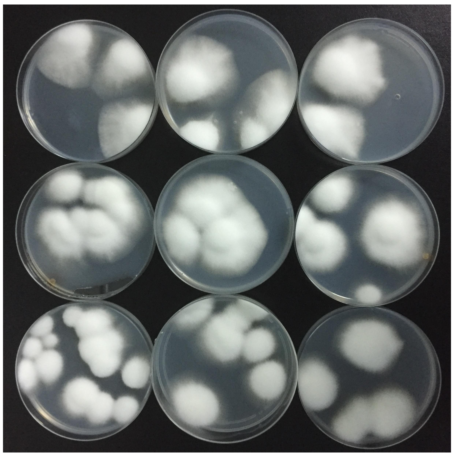

24 h after administration

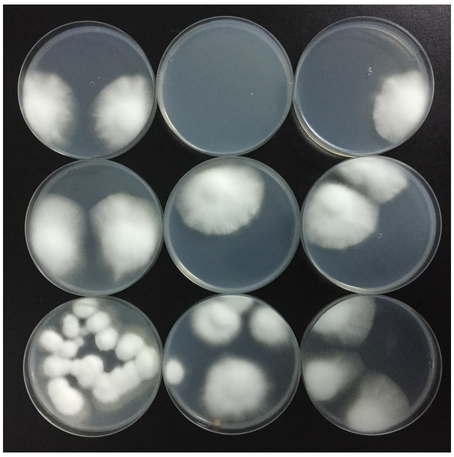

48 h after administration

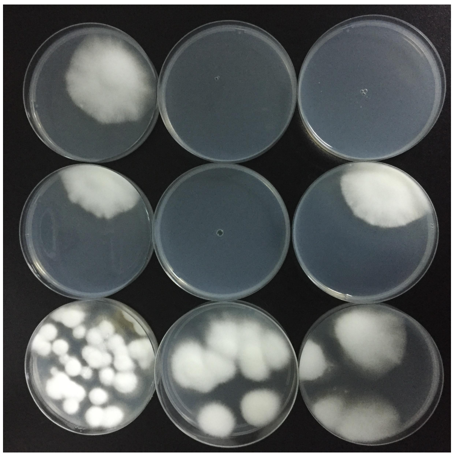

72 h after administration

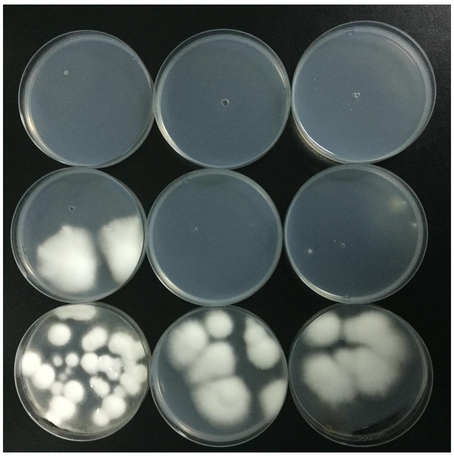

96 h after administration

Figure S1. Colonies of different time points in Time-Kill Curve
